# Daily persistent headache after a viral illness during a worldwide pandemic may not be a new occurrence: Lessons from the 1890 Russian/Asiatic flu

**DOI:** 10.1177/0333102420965132

**Published:** 2020-11-04

**Authors:** Todd D Rozen

**Affiliations:** Department of Neurology, Mayo Clinic, FL, USA

**Keywords:** New daily persistent headache, viral pandemic, Russian flu, SARS-CoV-2, neurasthenia, headache

## Abstract

New daily persistent headache was first documented in the medical literature in the 1980s. The leading trigger is a viral illness. As we navigate our way thru the current SARS-CoV-2 pandemic, looking back at past viral epidemics may help guide us for what to expect in the near future in regard to headaches as a persistent manifestation of the SARS-CoV-2 infection. The 1890 viral pandemic known as the “Russian or Asiatic flu”, has extensive documentation about the neurologic sequelae that presented months to years after the pandemic ended. One of the complications was daily persistent headache. There are actually many similarities between the viral presentation of the 1890 pandemic and the current SARS-CoV-2 pandemic, which may then suggest that not only will NDPH be part of the neurological sequelae but a possible key consequence of the SARS-CoV-2 infection.

New daily persistent headache (NDPH) has become one of the most challenging disorders in headache clinics. The leading triggering event for NDPH is a viral illness, which is noted in upwards of 30% of cases (1–3). The infectious trigger is equally prevalent in both sexes (4). As we navigate our way through the current SARS-CoV-2 pandemic, a possible increase in post-viral NDPH cases could be forecast. Already there are reports of headache being a primary symptom of acute SARS-CoV-2 infection and a predominant lingering complaint of the prolonged illness now being reported in patients with even mild cases of SARS-CoV-2 (5–7). Looking back at past viral epidemics may help guide us for what to expect in the near future.

NDPH was first designated as a distinct headache condition in 1986 (8). Before Vanast’s (8) original description of NDPH, no one had denoted a specific headache syndrome that started daily from onset. However, there are clues from the medical literature of the 19th century that indeed NDPH existed, at least the post-infectious subtype. The worst known viral pandemic to date was the 1918 “Spanish influenza”, which killed between 50 and 100 million people (9). Surprisingly, there is very little written about the “headache” manifestations of the infection beyond the acute state of the illness. The 1890 viral pandemic, however, known as the “Russian or Asiatic Flu”, which killed one million individuals, actually has extensive documentation about the neurologic sequelae that presented months to years after the pandemic ended (10). The pandemic occurred in three waves from 1889–1892 and was possibly caused by the influenza A subtype H3, but the true viral pathogen is unknown to this date (11). One of the primary neurologic manifestations of the infection was the controversial syndrome of neurasthenia, or nerve exhaustion. The hallmarks of this were headache, lethargy and insomnia (12). Neurasthenia has now been recognized as a probable offshoot of chronic fatigue syndrome/myalgic encephalomyelitis and/or fibromyalgia, but some of these cases appear to be post-infectious NDPH (13). Interestingly, the same symptom complex is now being noted in patients with the prolonged illness variant of SARS-CoV-2 (7).

## Did NDPH occur during previous viral pandemics?

Headache was one of the primary manifestations of having influenza during the 1890 pandemic. Reports from Europe noted head pain as a complaint in 75–83% of sufferers in the acute stage of the illness (14). How many of these individuals developed into true ICHD-defined NDPH is unknown, but when looking at the published accounts, both in case series and in books, a number of sufferers did continue with a daily persistent headache that was present months to years after their initial infection. Althaus (15), in his book on influenza from 1891, noted that there was “an immense variety of severe symptoms not only during the primary attack but also in many cases for a long time subsequently”. He also opined that the “number of nervous sequels which appeared after grip (influenza) was largely in excess of other post febrile neuroses”. He points out that the headache of influenza “generally last two or 3 days; yet it may continue for 2 to 3 weeks or more”. Byrne (16), in an article in *JAMA* from 1900, noted that in students from St. Charles College in the UK that their “troubles continued three or four months after the acute attack of influenza had passed and these included head troubles, most often in the occipital, sometimes in the temporal regions and rather odd sensations about the head other than pains or aches”. 

As stated, one of the main neurologic sequelae of influenza was the syndrome of neurasthenia. Typically, the neurasthenic symptoms would start along with or just after resolution of the acute phase of influenza and could last for months to years after the onset of the infection (12,17). Multiple treatises were written on this condition from the late 1890s through the mid-1900s. In Cobb’s (12) text from 1920, he remarked that the majority of individuals with neurasthenia give a history of this “dread disease, often in a severe form. It [influenza] is so common a precursor that we can say that this malady maybe regarded as an extremely frequent and potent source of Neurasthenia.” He points to the 1890 pandemic as the first time influenza was connected to neurasthenia, or post-influenza debility as it was also called. The American George Beard (18) in 1869 first coined the term neurasthenia, but Cobb (12) remarked that Beard did not note influenza as an etiologic factor for the syndrome, possibly because influenza was more frequent in Europe. Cobb’s (12) text on neurasthenia actually devoted an entire section to headache. He noted several varieties of headache and three were occurring on a daily basis: “We may fairly regard headache as a more constant symptom… One type remains constant throughout the day, with the single exception that it becomes worse after meals and remains bad at such times for anything up to an hour. Another type is present on waking, remains constant until the middle of the morning; improves until lunch, and suffers an exacerbation after the midday meal; improves in the late afternoon, and again becomes troublesome in the evening”. A third type was only severe upon awakening and then improving after arising “its worst form in the early morning and improves after the early cup of tea or the first meal and then disappears for the rest of the day” (12). Proust and Ballet (17), in their text on neurasthenia, also remarked on how a subset of patients had headache which was “continuous and incessant”. All of these descriptions could possibly reach ICHD-3 criteria for NDPH as they began daily from onset and lasted for more than 3 months (19).

## Headache location

“Classic” NDPH-based head pain is bilateral, constant and of moderate to severe intensity (1,2). Location can be anywhere on the head but a majority have some occipitonuchal-based pain, while retro-orbital discomfort occurs in almost 50% of sufferers (1). What is not known at present is if different subtypes of NDPH, based on triggering events, have a diverse presentation for head pain location (4). The various locations of the chronic headache noted by observers in their patients after the 1890 viral pandemic included: Helmet like, skull cap, a band stretched across the head and boring pain in the occipital region or nape of the neck (12,17). The quality was described as: Throbbing, sharp, burning, emptiness and/or pressure. Migrainous associated symptoms and headache-related disability, which can occur with NDPH, are also referred to in older texts: “All intellectual work, reading, composing a letter, a conversation however short, emotion, noise, all augment the headache” (1,2,17).

## Hypermobility

The majority of NDPH sufferers have hypermobility issues (20). Recent studies in patients with Ehlers-Danlos syndrome (the most common form of hypermobility) have demonstrated an alteration in inflammatory/pain modulation genes including CFD, AQP9, COLEC12, KCNG5, PRLR; thus, a possible predisposing factor for an abnormal immune response in the hypermobile population to infections (21). Fascinatingly, sufferers who developed chronic daily headache after the 1890 viral pandemic may also have demonstrated hypermobility. Bouveret (22), Cobbs (12) and others noted two physical subtypes who suffered from post influenza debility. One group was tall and thin and who had low blood pressure, while a second group was well-fed and robust. The former group was the one who developed frequent headaches, lethargy, insomnia and had signs of hyperalgesia on exam (“any pain he suffers is out of proportion to its cause”) after the viral infection (12). Thus, there is the tall, lanky appearance that is noted in the majority of the hypermobile NDPH patients and their predisposition for hypotension and autonomic dysfunction, which is a hallmark of patients with joint hypermobility/Ehlers-Danlos syndrome (20,23). As high cervical spine irritation can be a major contributor of NDPH in hypermobile patients (with and without an infectious trigger), it is interesting to note that a large majority of patients who developed headache during the “Russian flu” not only had occipital-based pain but also neck pain. In a report by Eade (24) from his observation of patients from England during the 1890 pandemic, he noted “a peculiar chronic affection of portions of the spinal column, in the form of persistent tenderness of some of the vertebras, most commonly of the cervical and dorsal regions”. One could hypothesize that the manifestations of the influenza, including coughing, sneezing, rigors with fever and prolonged periods of bed rest, could irritate the high cervical facet joints, leading to trigemino-cervical complex activation and persistent head and neck pain. To bring this back to our current situation with SARS-CoV-2, Eade (24) also noted in the influenza-afflicted patients “a varying amount of loss of taste or smell has been frequently noted”. Thus, a common connection is noted between past and current viral pandemics, and possibly suggests a harbinger of things to come with regard to headache-related issues with SARS-CoV-2.

## Conclusion

No one can predict how many sufferers of the current SARS-CoV-2 pandemic will develop NDPH, but headache is one of the most common symptoms of the acute infection and appears to be part of the symptom complex of the newly documented prolonged illness associated with the virus. As there are similarities between the viral presentation of the 1890 pandemic and the current SARS-CoV-2 pandemic, this may suggest that not only will NDPH be part of the neurological sequelae but a possible key consequence of the SARS-CoV-2 infection.

## Clinical implications

- The most common triggering event of NDPH is a viral illness.
- History may suggest that NDPH has been a sequela of prior viral pandemics including the 1890 Russian or Asiatic flu.
- There are reports of headache being a primary symptom of acute SARS-CoV-2 infection and a lingering complaint of the prolonged illness.
- Similarities between the present and past viral pandemics may suggest that NDPH will be part of the neurological sequelae of the SARS-CoV-2 infection.
